# LGALS3 Is a Poor Prognostic Factor in Diffusely Infiltrating Gliomas and Is Closely Correlated With CD163+ Tumor-Associated Macrophages

**DOI:** 10.3389/fmed.2020.00182

**Published:** 2020-05-21

**Authors:** Wan-Ming Hu, Yuan-Zhong Yang, Tian-Zhi Zhang, Chang-Fei Qin, Xue-Nong Li

**Affiliations:** ^1^Department of Pathology, School of Basic Medical Sciences, Southern Medical University, Guangzhou, China; ^2^Department of Pathology, Sun Yat-sen University Cancer Center and State Key Laboratory of Oncology in South China and Collaborative Innovation Center for Cancer Medicine, Guangzhou, China; ^3^Department of Pathology, The Seventh Affiliated Hospital, Sun Yat-sen University, Shenzhen, China

**Keywords:** LGALS3, glioblastoma, prognosis, TAM, IDH, 1p/19q, TERT

## Abstract

**Background:** Glioma, the most common brain tumor, is a heterogeneous group of glia-derived tumors, the majority of which have characteristics of diffuse infiltration and immunosuppression. The LGALS protein family is a large class of sugar-binding proteins. Among them, LGALS3 has been reported to promote tumor development and progression in some cancers. However, the clinical significance and biological functions of LGALS3 in glioma remain virtually unknown. The purpose of our research is to detect LGALS3 expression and its prognostic value in glioma and reveal the relationship between its expression and the clinico/molecular-pathological features of patients and immune cell infiltration.

**Methods:** LGALS3 protein expression was examined by immunohistochemistry. The mRNA expression data of LGALS3 was downloaded and analyzed from TCGA and Rembrandt datasets. The association between LGALS3 and glioma clinically relevant diagnostic/molecular markers (IDH, 1p19q, ATRX, MGMT, and TERT) was examined using the Chi-Squared (χ^2^) test. The correlation between LGALS3 expression and the infiltration of multiple intra-tumoral immune cell types, including B cells (CD20), T cells (CD4 and CD8), macrophages (CD68), and M2 tumor-associated macrophages (CD163), was evaluated by Spearman correlation analysis. Kaplan-Meier analysis and the Cox regression analysis were applied to evaluate the prognostic value of LGALS3 in glioma. The log-rank test was used to evaluate Kaplan-Meier results for significance.

**Results:** Out of all 304 glioma cases, LGALS3 protein was expressed in 125 glioma cases (41.1%, 125/304), with 69.2% (9/13) in WHO I, 9.8% (8/82) in WHO II, 34.2% (26/76) in WHO III, and 61.7% (82/133) in WHO IV. The expression of LGALS3 was correlated with patient age, WHO grade, PHH3 (mitosis), Ki67 index, IDH, 1p/19q codeletion, and TERT promoter status. LGALS3 was an independent poor prognostic marker in diffusely infiltrating gliomas and was positively correlated with immune cell infiltration, particularly CD163+ tumor-associated macrophages in the TCGA dataset, Rembrandt dataset, and our SYSUCC cohort (*R* = 0.419, 0.627, and 0.724).

**Conclusion:** LGALS3 was highly expressed in pilocytic astrocytoma, GBM, and IDH wild-type LGG. It served as a poor prognostic marker in diffusely infiltrating gliomas. Based on its prognostic significance and strong correlation with CD163+ TAMs, it may act as an important therapeutic target for human glioma.

## Introduction

Glioma is a heterogeneous group of brain neoplasms originating from glial cells, which exhibit a wide range of aggressiveness depending on subtype and grade. Gliomas comprise ~30% of all brain tumors and central nervous system tumors and 80% of all malignant brain tumors (1). The incidence is five per 100,000 to eight per 100,000 (2, 3). According to the characteristics of specific cases, the World Health Organization divides gliomas into four different grades (WHO grade I-IV): pilocytic astrocytoma (WHO I) is a borderline tumor with generally circumscribed slow growth; diffuse astrocytoma/oligodendroglioma (WHO II) and anaplastic astrocytoma/oligodendroglioma (WHO III), which are collectively known as lower grade glioma (LGG); and glioblastoma (GBM, WHO IV), which is the most aggressive type of brain tumor. Both LGG and GBM are diffusely infiltrating gliomas with malignant biological behaviors (4). Despite improvements in treatment and extensive research into the molecular mechanism of the occurrence and development of glioma, the overall prognosis of diffusely infiltrating glioma, especially GBM, is still extremely poor.

In recent years, tumor-associated macrophages (TAMs) have been shown to participate in glioma progression and have attracted increasing attention. Therefore, the discovery of novel biomarkers associated with TAMs of glioma will facilitate the screening of high-risk patients who may benefit from more targeted clinical interventions.

Members of the LGALS protein family have a carbohydrate recognition domain (CRD) of 135 amino acid homologous sequences. Hitherto, 15 LGALS proteins have been identified. LGALS proteins are involved in many cellular biological processes both inside and outside the cell. In the LGALS family, LGALS3 has a special domain that recognizes and binds to β-galactosides on cellular glycoproteins and glycolipids (5). LGALS3 may be observed in the cytoplasm and in the nucleus as well as the extracellular matrix (6). It serves different biological functions, such as cell growth, cell adhesion, angiogenesis, and apoptosis (7).

LGALS3 can be expressed in different types of tumors, and accumulating evidence has proved that LGALS3 plays a vital role in tumorigenesis and development (6, 8–16). Recently, a study indicated that LGALS3 can promote the therapeutic resistance of glioblastoma and is related to tumor risk and prognosis (17). However, its prognostic significance needs to be further confirmed in large glioma samples, and, hitherto, no studies have explored the role of LGALS3 in the glioma immune microenvironment and its correlation with key molecular markers, including isocitrate dehydrogenase 1 (IDH1), alpha-thalassemia/mental retardation X-linked (ATRX), O-6-methylguanine-DNA methyltransferase (MGMT), telomerase reverse transcriptase (TERT), and 1p19q.

## Materials and Methods

### Patients and specimens

Formalin-fixed paraffin-embedded tissues were obtained from the archives of the Department of Pathology, Cancer Center, Sun Yat-sen University, Guangzhou, China, between 2008 and 2016, including 304 glioma patients of WHO grade I–IV. Written informed consent was acquired for all samples prior to this study, the protocols for this research were approved by the Scientific Ethics Committee of the Sun Yat-sen University Cancer Center (SYSUCC), and all methods were carried out in accordance with relevant guidelines and regulations. The official approval by the committee was waived, as this is a retrospective study using archived tissue samples. The choice of samples was based on the several factors: the availability of resected tissue, the accuracy of follow-up data, and no radiotherapy or chemotherapy before surgery. The cohort included 13 cases of pilocytic astrocytoma (WHO I), 82 cases of oligodendroglioma and diffuse astrocytoma (WHO II), 76 cases of anaplastic oligodendroglioma and anaplastic astrocytoma (WHO III), and 133 cases of glioblastoma (WHO IV) with complete molecular information (IDH, 1p/19q, ATRX, MGMT, and TERT). Overall survival is the time interval between the date of diagnosis and the date of final death or the last known follow-up.

### TCGA and Rembrandt Datasets

The glioma sample expression profiles and detailed clinical information were downloaded from the TCGA and Rembrandt glioma cohort public websites (https://tcga-data.nci.nih.gov/tcga/tcgaDownload.jsp) and (http://rembrandt.nci.nih.gov). The Gravendeel dataset was downloaded from GlioVis (http://gliovis.bioinfo.cnio.es/).

Patient characteristics in SYSUCC, TCGA, and Rembrandt datasets were shown in Tables S2, S3. TMA, IHC, and molecular detection of IDH, ATRX, MGMT, TERT, and 1p/19q were performed as described previously (18, 19). Mouse monoclonal to human anti-LGALS3 antibody was used for IHC (Zsbio, China, clone number: OTI15E4). Antibodies used for detecting immune cell types were CD4 (Zsbio, China, clone number: UMAB64), CD8 (Zsbio, China, clone number: SP16), CD20 (Zsbio, China, clone number: OTI4B4), CD68 (Zsbio, China, clone number: KP1), and CD163 (Zsbio, China, clone number: 10D6).

### Scoring of Staining

LGALS3 is mainly expressed in cytoplasm of glioma cells. IHC scoring criteria were established by combining the positive proportion (1 for 0–25%, 2 for 26–50%, 3 for 51–75%, and 4 for >75%) and staining intensity (0 for no staining, 1 for light yellow, 2 for yellowish brown, and 3 for brown) of the stained tumor cells. The final LGALS3 IHC scores were obtained using the following formula: final immunohistochemistry score (0–12) = values of intensity (0–3) × values of percentage counts (0–4). We then used ROC curves to find the best cut-off values of the LGALS3 score with respect to patient status and OS. Base on the best cut-off value “2” found by ROC curve (Figure S1 and Table S1), a two-level grade system of LGALS3 staining was quantified: 0–2 indicates negative and 3–12 indicates positive. The number of immune cells (CD20+ B cells, CD4+ T cells, CD8+ T cells, CD68+ macrophages, and CD163+ tumor associated macrophages) was assessed at high power magnification (×400). For each antibody, the mean number of labeled cells per high-power field was calculated as the total number of positive cells in five randomly selected tumor regions divided by five. All samples were scored separately by two independent neuropathologists, who were blinded to the patient data, and the final scores were averaged for further comparative evaluation.

### Statistical Analysis

Associations between LGALS3 and clinical/molecular variables were evaluated by use of 2 × 2 contingency tables and the Chi square (χ^2^) test. Survival curves and the Kaplan-Meier estimator were computed and plotted. Survival differences according to LGALS3 expression were analyzed by the log-rank test. Univariate and multivariate Cox regression analyses were used to assess the influence of variables on survival. The correlation between LGALS3 expression and the numbers of multiple intra-tumoral immune cell types was determined using the Spearman correlation coefficient. Other statistical details are displayed in the Supplementary Materials. All statistical analyses were performed with SPSS (version 16.0, Chicago, USA), and significance was defined as *p* < 0.05.

## Results

### LGALS3 was Mainly Expressed in Pilocytic Astrocytoma, GBM, and IDH Wild-Type LGG

**Figure 1:**
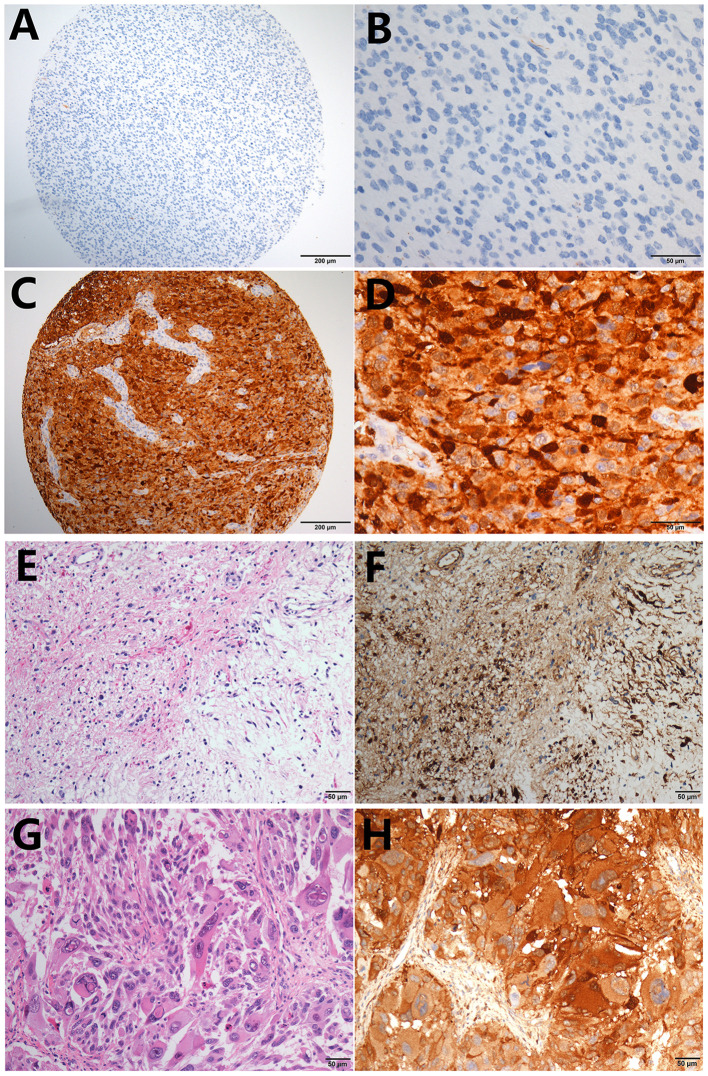
Typical immunohistochemistry results of LGALS3. **(A)** LGALS3 negative case from an anaplastic astrocytoma, IDH mutated (X100), **(B)** negative case of high-power field X400, **(C)** LGALS3 positive case from an anaplastic astrocytoma, IDH wildtype (X100), and **(D)** positive case of high-power field X400. Pilocytic astrocytoma showed diffuse strong positive **(E)** HE X400 and **(F)** IHC X400. GBM also showed strong and diffuse LGALS3 immunoreactivity **(G)** HE X400 and **(H)** IHC X400.

LGALS3 was mainly expressed in cytoplasm, and weak expression in endothelial cells was used as an internal control in glioma. The typical positive and negative results of IHC staining for LGALS3 in glioma are presented in Figures 1A–D. Distinctly high expression of LGALS3 was observed in pilocytic astrocytoma and GBM, both with a diffuse pattern (Figures 1E–H). In total, out of all 304 glioma cases, LGALS3 protein expression was positive in 125 glioma cases (41.1%, 125/304), with 69.2% (9/13) in WHO I (pilocytic astrocytoma), 9.8% (8/82) in WHO II (diffuse astrocytoma and oligodendroglioma), 34.2% (26/76) in WHO III (anaplastic astrocytoma and oligodendroglioma), and 61.7% (82/133) in WHO IV (glioblastoma) (Figure S2A). Further analysis showed that LGALS3 was mainly expressed in IDH wildtype LGG compared with IDH mutated LGG (Figure S2B).

### LGALS3 Correlated With Patient Age, WHO Grade, Mitosis Figure (PHH3), Ki67, IDH, TERT, and 1p/19q, and Served as an Independent Poor Prognostic Factor in Diffusely Infiltrating Glioma

**Figure 2:**
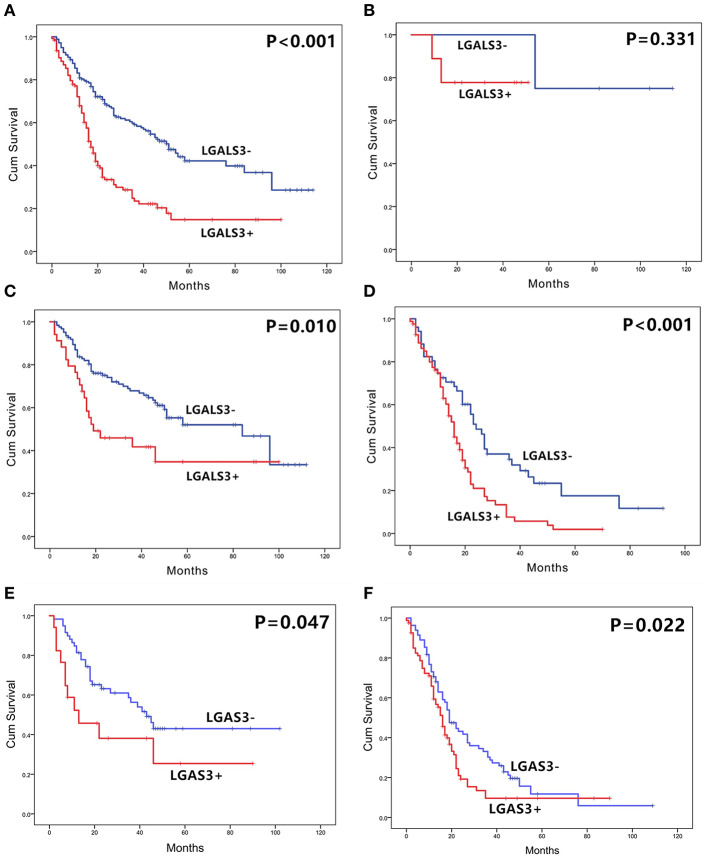
The overall survival curves according to LGALS3 expression. **(A)** All the glioma cases of WHO I-IV, **(B)** WHO I PA, **(C)** WHO II-III LGG, **(D)** WHO IV GBM, **(E)** WHO III anaplastic astrocytoma, and **(F)** WHO II-IV IDH wildtype glioma. LGALS3-positive patients presented with significantly shorter OS than those of negative patients in all the gliomas, LGG, GBM, AA, and IDH glioma but no statistical difference in PA. PA, pilocytic astrocytoma; LGG, lower grade glioma; GBM, glioblastoma; AA, anaplastic astrocytoma.

The correlation between LGALS3 expression and the clinicopathological characteristics of glioma patients is shown in Table 1. LGALS3 was significantly associated with patient age (*p* < 0.001), WHO grade (*p* < 0.001), IDH status (*p* < 0.001), 1p/19q codeletion status (*p* = 0.007), TERT promoter status (*p* = 0.016), PHH3 (*p* < 0.001), and the Ki67 index (*p* = 0.002). Kaplan-Meier plots revealed that LGALS3 expression was a significantly unfavorable prognostic marker in diffusely infiltrating gliomas (WHO II-IV) but not in pilocytic glioma (WHO I). LGALS3 -positive patients had a worse median survival than LGALS3-negative patients (20 vs. 35 months, *P* < 0.001) in diffusely infiltrating gliomas. Further stratification analysis revealed that LGALS3 was also a poor prognostic marker in LGG, GBM, anaplastic astrocytoma, and IDH wild-type glioma (Figure 2). Univariate analysis revealed that old patient age (*P* = 0.006), high WHO grade (*p* < 0.001), high PHH3(mitosis figures) (*p* = 0.002), high Ki67 index (*p* < 0.001), TERT promoter mutation (*p* < 0.001), and positive expression of LGALS3 (*p* < 0.001) were negatively correlated with the prognosis of all 291 diffusely infiltrating glioma patients, while IDH mutation (*p* < 0.001), 1p/19q codeletion (*p* = 0.001), and MGMT promoter mutation (*p* < 0.001) were good prognostic indicators. Multivariate Cox analysis confirmed that LGALS3 was an independent risk factor for survival in diffusely infiltrating glioma with an adjusted HR of 1.537 (95% CI: 1.204–1.987, *p* = 0.031, Table 2).

**Table 1:** Clinical-pathological characteristics of the glioma patients and LGALS3 expression.

	**No. of cases**	**LGALS3**		***P***
		**Negative**	**Positive**	
**Age**				**0.001**
≤ 45	163	110	53	
>45	141	69	72	
**Gender**				0.237
Male	180	101	79	
Female	124	78	46	
**WHO grade**				**<0.001**
Low (I+II)	95	78	17	
High (III+IV)	209	101	108	
**Location**				0.615
Supratentorial	287	168	119	
Subtentorial	17	11	6	
**IDH**				**<0.001**
Wildtype	177	76	101	
Mutated	127	103	24	
**ATRX**				0.562
Wildtype	160	97	63	
Mutated	144	82	62	
**1p/19q**				**0.002**
Co-deleted	48	38	10	
Intact	256	141	115	
**TERT**				**0.003**
Wildtype	160	107	53	
Mutated	144	72	72	
**MGMT**				0.541
Promoter methylated	159	91	68	
Promoter Unmethylated	145	88	57	
**P53**				0.589
≤ 10%	110	67	43	
>10%	194	112	82	
**PHH3**				**<0.001**
≤ 5	123	95	28	
>5	181	84	97	
**Ki67**				**<0.001**
≤ 10%	74	57	17	
>10%	230	122	108	

*Bold values represent p < 0.05*.

**Table 2:** Univariate and multivariate analyses for overall survival.

**Variable**	**Univariate analysis**		**Multivariate analysis**	
	**Hazard ratio (95% CI)**	***P*-value**	**Hazard ratio (95% CI)**	***P*-value**
Gender: (Male)	1.123 (0.806–1.565)	0.493		
Age: (years>45)	**1.579 (1.143–2.181)**	**0.006**		
Location: (Supratentorial)	1.126 (0.551–2.298)	0.745		
WHO Grade: (High)	**2.643 (1.750–3.992)**	**<0.001**	**2.270 (1.453–3.547)**	**<0.001**
IDH: (Mutated)	**0.347 (0.238–0.506)**	**<0.001**	**0.606 (0.393–0.934)**	**0.023**
1p/19q: (Co-deleted)	**0.307 (0.156–0.603)**	**0.001**	**0.267 (0.124–0.572)**	**0.001**
ATRX: (Mutated)	0.734 (0.529–1.017)	0.063		
TERTp: (Mutated)	**1.921 (1.380–2.675)**	**<0.001**	**2.015 (1.412–2.875)**	**<** **0.001**
MGMTp: (Methylated)	**0.525 (0.377–0.731)**	**<0.001**	**0.450 (0.320–0.633)**	**<** **0.001**
P53: (>10%)	1.249 (0.886–1.762)	0.204		
PHH3: (>5)	**1.735 (1.227–2.454)**	**0.002**		
Ki67: (>10%)	**2.293 (1.444–3.639)**	**<0.001**		
LGALS3: (High)	**1.846 (1.331–2.561)**	**<0.001**	**1.537 (1.204–1.987)**	**0.031**

*Method = Forward Stepwise (Conditional LR). Bold values represent p < 0.05*.

### LGALS3 was Positively Correlated With Immune Cell Infiltration in Glioma, Particularly CD163+ TAMs

**Figure 3:**
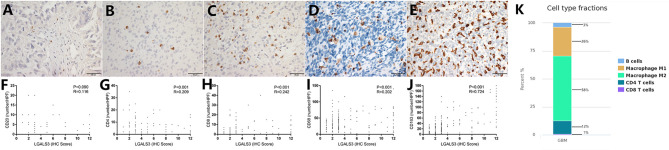
Representative IHC images of immune cell markers [CD20 **(A)**, CD4 **(B)**, CD8 **(C)**, CD68 **(D)**, and CD163 **(E)**] expression and its correlation with LGALS3 expression in human glioma specimens (X400). Scatterplot showed significant positive correlation between immune cells numbers and LGALS3 expression. **(F)** CD20+ B cells were not correlated with LGALS3 expression, but significant positive correlation was found between CD4+ T cells **(G)**, CD8+ T cells **(H)**, CD68+ macrophages **(I)**, CD163+ TAMs **(J)**, and LGALS3 expression. **(K)** TCIA (The Cancer Immunome Atlas) database query results of different immune cell type in glioma: macrophages are the predominant infiltrating immune cell in glioma.

In contrast to other tumors, lymphocytic infiltration was minimal in glioma. Traditionally, blood-brain barriers impaired the ability of peripheral lymphocytes to transport to the glioma microenvironment, and gliomas have been classified as “cold tumors” with very little lymphocyte infiltration. We found only few CD20+ B lymphocytes and CD4+/CD8+ T lymphocytes in gliomas, both of which occurred primarily within perivascular spaces and around areas of necrosis. However, most glioma cases showed CD68+ macrophage/microglia and CD163+ TAM infiltration. TCIA (The Cancer Immunome Atlas) database also confirmed our results (Figure 3K). Specifically, CD20+ B lymphocytes (range: 0–20/HPF) were detected in 69/304 (22.7%) cases. CD4+ T lymphocytes (range: 0–35/HPF) were detected in 115/304 (37.5%) cases, CD8+ T lymphocytes (range: 0–40/HPF) were detected in 103/304 (33.9%) cases, CD68+ macrophages (range: 0–150/HPF) were detected in 294/304 (96.7%) cases, and CD163+ TAMs (range: 0–180/HPF) were detected in 275/304 (90.5%) cases (Figures 3A–E) in our cohort. The median number of CD163+ TAMs was 20/HPF (range: 0–180/HPF), with a mean number of 28/HPF (Table 3). Spearman correlation analysis revealed that CD20+ B cells were not correlated with LGALS3 expression (Figure 3F), but significant positive correlations were found between the infiltration of CD4+ T cells (Figure 3G), CD8+ T cells (Figure 3H), CD68+ macrophages (Figure 3I), CD163+ TAMs (Figure 3J), and the expression of LGALS3. In addition, the number of CD163+ TAMs infiltration was strongly correlated with LGALS3 (*R* = 0.724) in our cohort (Figure 3J).

**Table 3:** Quantification of immune cells (CD20+ B cells, CD4+ T cells, CD8+T cells, CD68+ Macrophages, and CD163+ TAMs) in our 304 glioma cases (WHO I–IV).

	**WHO I (*****n*** **=** **13)**		**WHO II (*****n*** **=** **82)**		**WHO III (*****n*** **=** **76)**		**WHO IV (*****n*** **=** **133)**		**Total (*****n*** **=** **304)**	
	**Median**	**IQR**	**Median**	**IQR**	**Median**	**IQR**	**Median**	**IQR**	**Median**	**IQR**
CD20	0	0–0	0	0–0	0	0–0	0	0–1	0	0–0
CD4	1	0–5	0	0–1	0	0–2	0	0–4	0	0–2
CD8	0	0–8	0	0–2	0	0–2	0	0–2	0	0–2
CD68	11	3–24	22	9–35	24	14–36	35	20–55	32	14–44
CD163	5	2–19	5	3–17	17	7–30	38	23–54	28	6–40

*IQR, interquartile range*.

### LGALS3 Expression and Prognostic Significance in Glioma Databases

**Figure 4:**
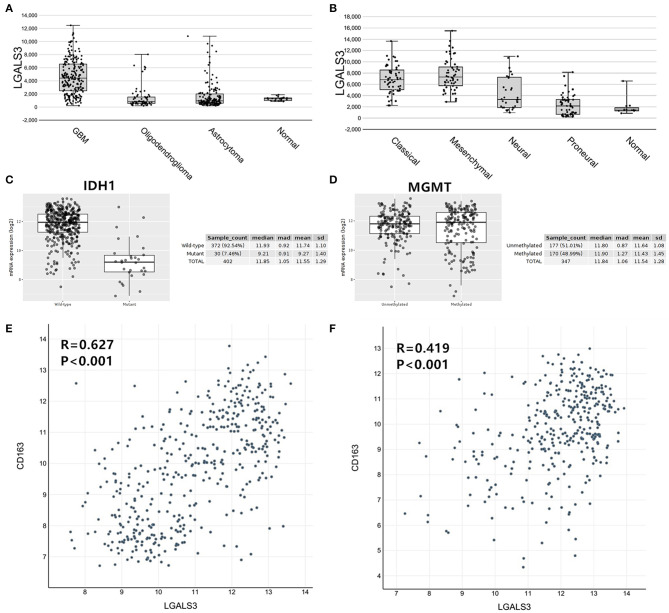
LGALS3 expression in TCGA and Rembrandt databases. **(A)** LGALS3 was highly expressed in TCGA GBMs, **(B)** particularly in classical and mesenchymal type. **(C)** LGALS3 mRNA was highly expressed in IDH wildtype glioma. **(D)** No statistical differences were found of LGALS3 expression in MGMT promoter mutation group and wildtype group. A scatterplot also showed significant positive correlation between CD163+ TAMs and LGALS3 expression in the TCGA **(E)** and Rembrandt datasets **(F)**, respectively.

**Figure 5:**
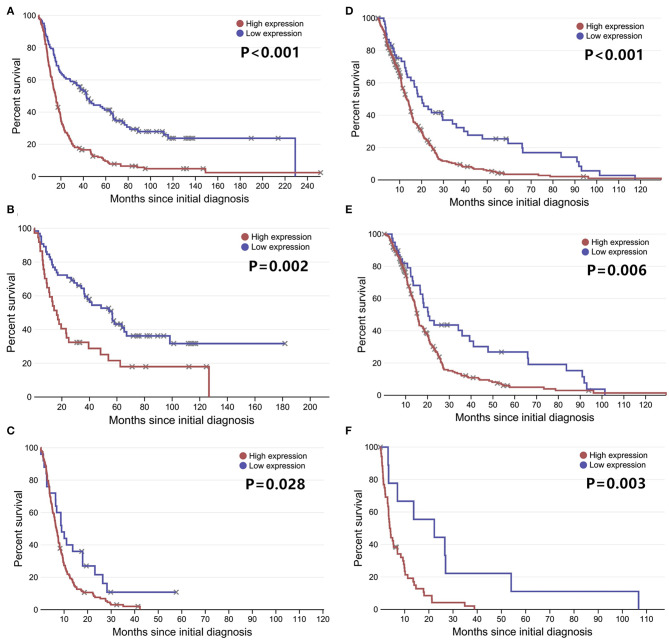
LGALS3 prognostic significance in TCGA and Rembrandt databases LGALS3 was a poor prognosis marker in Rembrandt glioma databases **(A)**, Rembrandt LGG databases **(B)**, Rembrandt GBM databases **(C)**, and TCGA GBM databases **(D)**. In the further analysis, LGALS3 has prognostic significance regardless of whether the patient receives chemotherapy **(E)** or not **(F)** in TCGA database.

To further confirm our results, we analyzed the expression of LGALS3 in two RNA-sequencing databases (TCGA and Rembrandt). Compared with that in other types of glioma and normal tissues, the expression of LGALS3 was also significantly higher in GBM and was mainly expressed in classical and mesenchymal GBM subtypes (Figures 4A,B). Similar to our results, further analysis indicated that LGALS3 mRNA was highly expressed in IDH wild-type glioma, but no significant difference was found between MGMT promoter mutation groups (Figures 4C,D). A significant positive correlation between CD163+ TAMs and LGALS3 expression was also found in the analysis of these two databases (Figures 4E,F). Regarding prognosis, Kaplan-Meier analysis also revealed that LGALS3 was a significant poor prognostic marker in diffusely infiltrating glioma, and it was a clear indicator of poor prognosis in both LGG and GBM regardless of whether chemotherapy was used (Figure 5).

**Figure 6:**
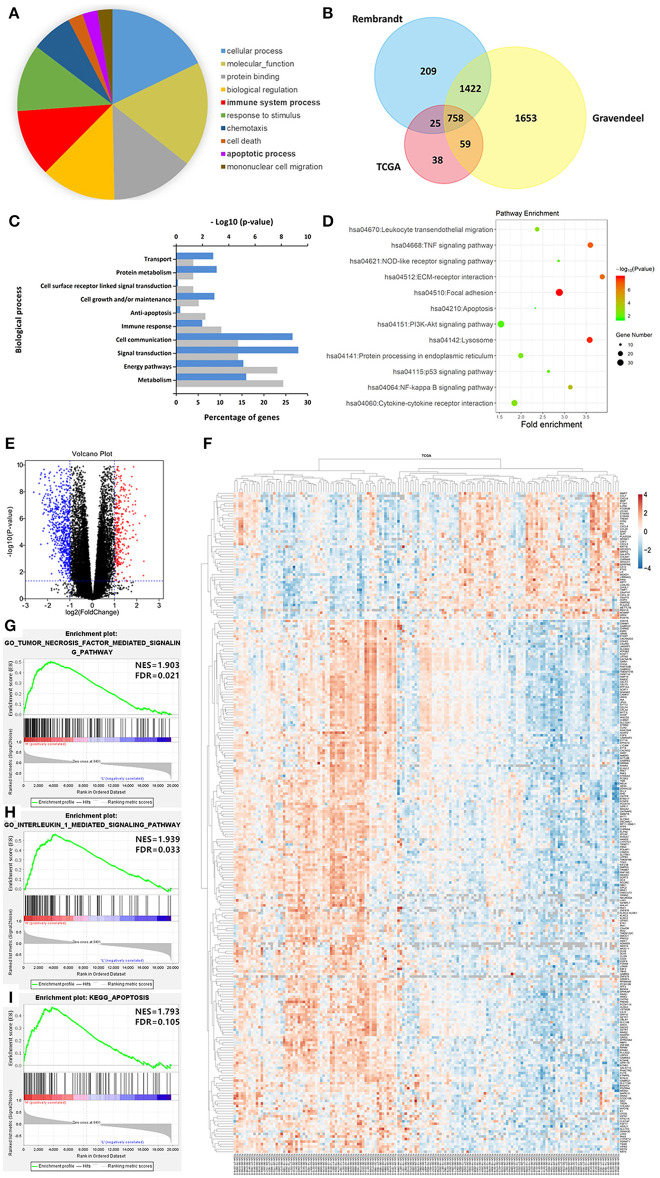
LGALS3-related biological signatures in glioma. **(A)** LGALS3-related biological processes in glioma using AmiGO2 (http://amigo.geneontology.org/amigo/landing). **(B)** Related genes of LGALS3 were chosen in glioma from the TCGA Rembrandt and Gravendeel datasets based on Pearson's correlation analysis (|*r*|≥ 0.3), and 758 intersection genes were screened out. **(C)** LGALS3 related biological process in glioma. **(D)** LGALS3 related KEGG signal pathways in glioma. **(E)** Different genes volcano plot in TCGA LGALS3 high and low group based on |logFC|>1 and *P* < 0.05. **(F)** Heatmap of partial differential genes in TCGA, CD163 was in the positively related gene list. **(G–I)** GSEA used to validate the different gene signatures, including the TNF signaling pathway, the IL1 signaling pathway, and apoptosis process.

To better understand the biological function of LGALS3 in glioma, we further researched genes correlated with LGALS3 expression in TCGA, Rembrandt, and Gravendeel databases using Pearson's correlation analysis (|*r*|≥ 0.3). A total of 758 genes were found at the intersection of the three datasets, including CD163 and other M2-related genes. Gene biological process analysis revealed that LGALS3 was associated with multiple biological processes including cell growth, apoptosis inhibition, and immune processes, and a KEGG pathway analysis revealed LGALS3 was involved in important inflammation and immune pathways including cytokine signaling, NF-kappaB, NOD-like receptor, and the TNF signaling pathway. We further analyzed the differentially expressed genes in TCGA between the LGALS3 high and low expression groups. In total, 1,084 differentially expressed genes (328 upregulated and 756 downregulated) were identified using the cutoff value of |logFC|>1 and *P* ≤ 0.05. GSEA analysis also validated the inflammation and immune gene signatures, including the TNF signaling pathway and IL1 signaling pathway (Figure 6). All these results indicated that LGALS3 may be involved in the immunosuppression in the glioma microenvironment by regulating the immune response and influencing the proportion of infiltrating immune cells (particularly TAMs).

## Discussion

In this study, we detected the expression of LGALS3 in a large number of glioma samples and found that LGALS3 was highly expressed in pilocytic astrocytoma and GBM. This finding was consistent with a previous report by Neder et al. (20), who considered LGALS3 protein to be an immunohistochemical marker to distinguish pilocytic astrocytoma from diffuse astrocytoma and glioblastoma from anaplastic oligodendroglioma. However, we think LGALS3 only has limited auxiliary diagnostic ability due to emerging molecular markers, such as IDH mutation, 1p/19q codeletion, and BRAF-KIAA1549 fusion (pilocytic astrocytoma's characteristic molecular alteration). Although GBM and pilocytic astrocytoma expressed LGALS3 with similar patterns, pilocytic astrocytoma is a distinct histologic and biologic subset of glioma that is characteristically slow-growing and non-infiltrative and targets totally different pathways compared with GBM. Due to the limited numbers of cases in our cohort, further research is needed to validate the significance of LGALS3 in larger PA cohorts.

We further found that LGALS3 expression was not only related to traditional clinicopathological factors, such as old age, high mitotic figures, and a high Ki67 index, but it was also related to important molecular markers (IDH, TERT promoter, and 1p/19q status). LGALS3 was mainly expressed in IDH wild-type glioma and was closely related to CD163+ TAMs. IDH wild-type status and high TAMs infiltration are two main factors indicating poor prognosis for glioma patients. This may explain why LGALS3 positive glioma patients have a significantly shorter OS than LGALS3 negative patients, suggesting that LGALS3 may play a role in malignant progression in glioma through changing the immune microenvironment in glioma. Some studies have also confirmed that LGALS3 played a key role in glioma development through increasing cell motility and invasion (21, 22). Vladimirova et al. (23) found that LGALS3 expression was mediated by Runx-2 transcription factors, which contributed to the malignant progression of glial tumors. Conversely, only Gordower et al. (24) reported that LGALS3 expression decreased as the WHO level increased in astrocytic tumors. We think the reason for this difference may be partially due to the small number of patient samples in their study. Moreover, online database analysis also verified our results. Patients with high expression of LGALS3 mRNA had a poor prognosis. LGALS3 was closely related to IDH status, CD163+ TAMs and was mainly expressed in IDH wild-type glioma. It is worth noting that LGALS3 mRNA was highly expressed in the mesenchymal subtype, a more malignant TCGA GBM subtype with a higher tendency for recurrence, metastasis, and increased vascularity.

Most importantly, we found that LGALS3 was involved in the regulation of the glioma immune microenvironment, particularly CD163+ TAMs. There is growing evidence that complex tumor microenvironments contribute to the malignant progression of gliomas (25, 26). Among the components of the tumor microenvironment, tumor-associated macrophages (TAMs) are considered to provide important support for tumor growth. Macrophages are divided into M1 and M2 subtypes according to their functions. Typically, CD68 is a general marker for macrophages, while CD163 is considered to be a highly specific marker for M2 type macrophages. CD68+ macrophages are usually activated during antigen presentation and inflammatory responses, while CD163+ macrophages have a large number of anti-inflammatory cytokines that contribute to immunosuppression and promote tumor development. Multiple studies have shown that TAMs can actively suppress adaptive immunity, promote tumor growth, and angiogenesis, and are very similar to M2 macrophages (27, 28). A previous study (29) confirmed that CD163+ TAMs played an important role in the biological process of glioma and that high expression of CD163 predicted poor prognosis in glioma patients. Another study suggested that the expression level of LGALS3 might affect macrophage infiltration in brain tumors (30), but only 16 GBM samples were used in their study. In the present study, we investigated the relationship between LGALS3 and TAMs in a large sample (304 glioma cases including 133 cases of GBM). We found that CD163+ TAMs were abundant in glioma, particularly in GBM and that LGALS3 was strongly correlated with the number of TAMs. GO and KEGG analyses also revealed that LGALS3 was involved in important inflammation and immune pathways, including cytokine signaling, NF-kappa B, NOD receptor, and the TNF signaling pathway. These results indicated that LGALS3 was involved in inflammatory and immune responses, which further contributed to malignant progression and shorter survival in glioma patients. However, the mechanism by which LGALS3 affects the glioma immune microenvironment and the exact pathways associated with LGALS3 in glioma need to be further explored in future studies.

In conclusion, we have shown that LGALS3 is a novel biomarker that is highly expressed in pilocytic astrocytoma, GBM, and IDH wild-type LGG. LGALS3 is associated with poor prognosis in diffusely infiltrating glioma and served as an important prognostic biomarker in LGG and GBM. Due to its strong correlation with immune cell infiltration in glioma, particularly the infiltration of CD163+ TAMs, it might be a novel and promising target and could provide a novel insight into potential therapeutic strategies for immune therapy in glioma.

## Data Availability Statement

Publicly available datasets were analyzed in this study. This data can be found here: https://tcga-data.nci.nih.gov/tcga/tcgaDownload.jsp, http://rembrandt.nci.nih.gov, http://gliovis.bioinfo.cnio.es/.

## Ethics Statement

Written informed consent was acquired from all samples before this study, and the protocols for this research were approved by the Scientific Ethics Committee of the Sun Yat-sen University Cancer Center (SYSUCC); all methods were carried out in accordance with relevant guidelines and regulations. The official approval by the committee was waived as this is a retrospective study using archived tissue samples.

## Author Contributions

W-MH was involved in the conception, design, and drafting the article. Y-ZY, T-ZZ, and C-FQ were involved in the analysis and interpretation of data. X-NL was involved in revising the article critically for important intellectual content.

## Conflict of Interest

The authors declare that the research was conducted in the absence of any commercial or financial relationships that could be construed as a potential conflict of interest.
