# Supplementary material for: LGALS3 Is a Poor Prognostic Factor in Diffusely Infiltrating Gliomas and Is Closely Correlated With CD163+ Tumor-Associated Macrophages
Source: Front Med (Lausanne). 2020 May 21;7:182. doi: 10.3389/fmed.2020.00182 (PMC7254797; doi:10.3389/fmed.2020.00182)
Supplement: Supplementary file 1 [file Data_Sheet_1.docx]

**Supplementary Methods:**
**Immunohistochemistry (IHC)**

IDH1-R132H (clone H09, 1:50; Dianova, Hamburg, Germany), ATRX (1:500; Sigma-Aldrich, St. Louis, MO, USA), and P53 /Ki67(1:100; Dako, Carpinteria, CA) was detected using formalin-fixed, paraffin-embedded tumor tissue sections on an automated Bench Mark Ultra (Ventana Medical systems, Roche, SW). Sections from known mutation-positive and immunoreactive tumors were used as positive controls. Negative controls consisted of sections incubated with Phosphate-buffered saline instead of the primary antibody.

**Molecular genetics**

1. Fluorescent in situ hybridization (FISH) was performed to detect 1p and 19q deletion using Vysis FISH Probe Kit (Abbott Molecular, Illinois, USA). At least≥25% of counted nuclei presented one target signal and two reference signals will be considered as 1p or 19q deleted when 100 non-overlapping nuclei were counted.

2. Mutation status of IDH1/2 and TERT promoter was studied with Sanger sequencing. Hotspot codons IDH1 Arg132 (exon 4)/IDH2 Arg172 (exon 4) and the hotspot mutations of TERT promoter at positions C228T and C250T were detected on an ABI® 3130 Genetic Analyzer (Life Technologies, USA), as described in another research [11].

3. The promoter methylation status of the MGMT gene was assessed using methylation-specific PCR with the EZ DNA Methylation Direct kit (Zymo Research Corp., Orange, California, USA).

**Statistic Methods:**

**Our own SYSUCC cohort (protein):**

Associations between LGALS3 and clinic/molecular variables were evaluated by use of 2 × 2 contingency tables and the Chi square (χ2) test.

Kaplan-Meier provides a method for estimating the survival curve, the log rank test provides a statistical comparison of two groups, and Cox's proportional hazards model allows additional covariates to be included. Survival curves and the Kaplan-Meier estimator were computed and plotted. Survival differences according to LGALS3 protein expression were analyzed by the log-rank test. Univariate and multivariate cox regression analyses were used to access the influence of variables on survival.

The correlation between LGALS3 expression and numbers of multiple intra-tumoral immune cell types used Spearman correlation coefficient.

**TCGA, Rembrandt and Gravendeel online database (mRNA):**

Comparison between two groups (IDH- vs IDH+; MGMT- vs MGMT+) was used independent sample T-test.

Survival curves and the Kaplan-Meier estimator were computed and plotted. Survival differences according to LGALS3 mRNA expression were analyzed by the log-rank test.

The correlation between CD163+ TAMs and LGALS3 mRNA expression was used Pearson correlation coefficient.

Genes correlated with LGALS3 mRNA expression in TCGA, Rembrandt and Gravendeel databases were found by using Pearson’s correlation analysis (|r|≥ 0.3).

**Supplementary Figures and Tables:**


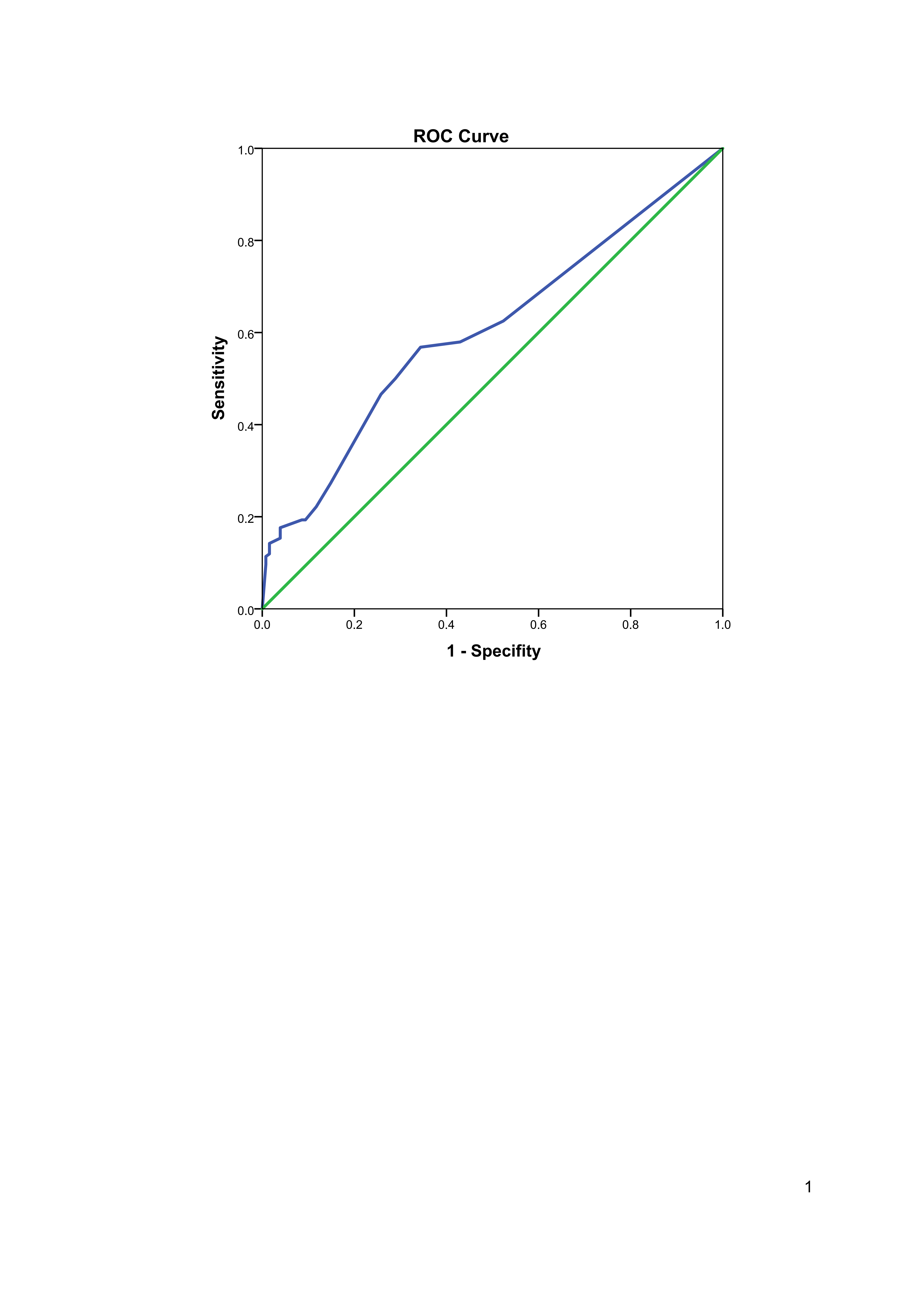


**Figure S1. Receiver-operator curve (ROC) were used to determine the cut-off score for positive expression of LGALS3 protein. (AUC = 0.666, 95%CI=0.542-0.668, P = 0.002)**

**Table S1. Coordinates of the curve**

| LGALS3 SCORE | Sensitivity | 1-Specifity | Specifity | Sensitivity+Specifity-1 |
| --- | --- | --- | --- | --- |
| 0 | 1.000 | 1.000 | 0.000 | 0.000 |
| 0.5 | 0.625 | 0.523 | 0.477 | 0.102 |
| 1.5 | 0.580 | 0.430 | 0.570 | 0.150 |
| **2** | **0.568** | **0.344** | **0.656** | **0.224** |
| 2.5 | 0.500 | 0.289 | 0.711 | 0.211 |
| 3 | 0.466 | 0.258 | 0.742 | 0.208 |
| 3.5 | 0.273 | 0.148 | 0.852 | 0.125 |
| 4 | 0.222 | 0.117 | 0.883 | 0.105 |
| 4.5 | 0.193 | 0.094 | 0.906 | 0.099 |
| 5 | 0.193 | 0.086 | 0.914 | 0.107 |
| 5.5 | 0.176 | 0.039 | 0.961 | 0.137 |
| 6 | 0.170 | 0.039 | 0.961 | 0.131 |
| 7 | 0.153 | 0.039 | 0.961 | 0.114 |
| 8 | 0.142 | 0.016 | 0.984 | 0.126 |
| 9 | 0.119 | 0.016 | 0.984 | 0.103 |
| 10 | 0.114 | 0.008 | 0.992 | 0.106 |
| 11 | 0.097 | 0.008 | 0.992 | 0.089 |
| 12 | 0.000 | 0.000 | 1.000 | 0.000 |

**Table S2. Patient characteristics of SYSUCC and TCGA**

|  | **SYSUCC** | **TCGA** |
| --- | --- | --- |
| **Sample(N)** | 304 | 669 |
| **Age (years)** | Median 42, range 6-78 | Median 47, range 14-89 |
| **Sex** | 180 male/124 female | 355 male/254 female  60 unknown |
| **Histology** |  |  |
| PA | 13 | 0 |
| A | 62 | 59 |
| O | 20 | 102 |
| AA | 44 | 116 |
| AO | 32 | 75 |
| GBM | 133 | 152 |
| Blank/NA | 0 | 165 |

PA=pilocytic astrocytoma; A=astrocytoma; O=oligodendroglioma; AA=anaplastic astrocytoma; AO=anaplastic oligodendroglioma; GBM=glioblastoma

**Table S3.** **Summary of the Rembrandt dataset.**

|  | **Clinical Attribute** | **Number of patients** | **% Of patients** |
| --- | --- | --- | --- |
| **Gender** | Male | 326 | 48.6% |
|  | Female | 177 | 26.4% |
|  | Blank/NA | 168 | 25.0% |
| **Disease Type** | GBM | 261 | 38.9% |
|  | Astrocytoma | 170 | 25.3% |
|  | Oligodendroglioma | 86 | 12.8% |
|  | Non tumor | 31 | 4.6% |
|  | Unknown | 68 | 10.1% |
|  | Unclassified | 1 | 0.1% |
|  | Mixed | 13 | 1.9% |
|  | Blank/NA | 41 | 6.1% |
| **WHO Grade** | I | 2 | 0.3% |
|  | II | 110 | 16.4% |
|  | III | 93 | 13.9% |
|  | IV | 140 | 20.9% |
|  | Blank/NA | 326 | 48.6% |
| **Race** | White | 433 | 64.5% |
|  | Black | 15 | 2.2% |
|  | Asian | 7 | 1.0% |
|  | Hispanic | 1 | 0.1% |
|  | Native Hawaiian | 3 | 0.4% |
|  | Unknown | 7 | 1.0% |
|  | Other | 5 | 0.7% |
|  | Blank/NA | 200 | 29.8% |


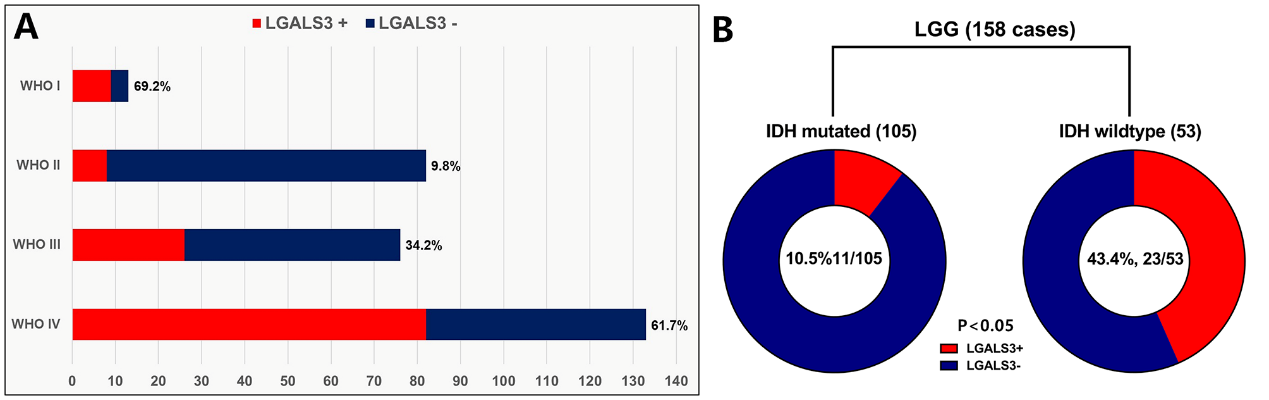


**Figure S2. Ratio of LGALS3 expression in glioma**.

(A)LGALS3 was significantly expressed in pilocytic astrocytoma (WHO I, 9/13, 69.2%) and in GBM (WHO IV, 82/133, 61.7%) (B) LGALS3 mainly expressed in IDH wildtype LGG (43.4% versus 10.5%, P<0.05)
