# Supplementary figures and images for: LGALS3 Is a Poor Prognostic Factor in Diffusely Infiltrating Gliomas and Is Closely Correlated With CD163+ Tumor-Associated Macrophages
Source: Front Med (Lausanne). 2020 May 21;7:182. doi: 10.3389/fmed.2020.00182 (PMC7254797; doi:10.3389/fmed.2020.00182)

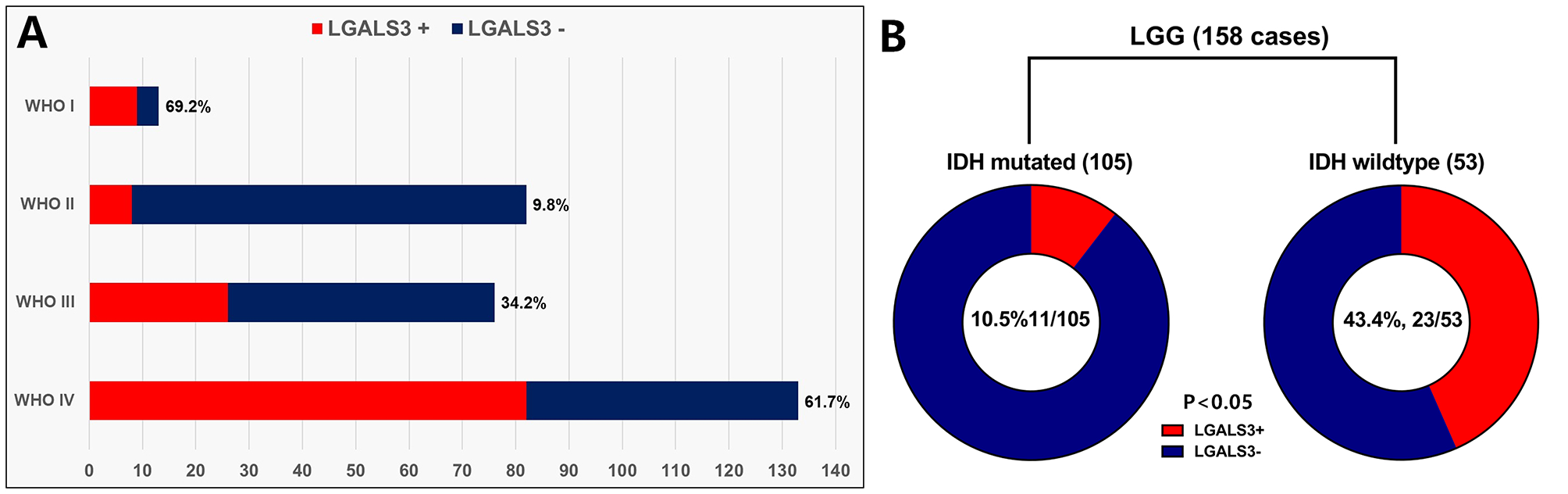

Supplement: Supplementary file 2 [file Image_1.TIF]
